# User Experience Comparison of a Traditional Mannequin-Based and an Augmented Reality Lumbar Puncture Simulator in Geriatric Medicine Training: Questionnaire Study

**DOI:** 10.2196/98090

**Published:** 2026-09-21

**Authors:** Emma Combret, Alexis Bourgeais, Frédéric Noublanche, Cédric Annweiler

**Affiliations:** 1 Laboratoire de Psychologie des Pays de la Loire (UR4638), Angers, Pays de la Loire, France; 2 Department of Geriatric Medicine and Memory Clinic, Research Center on Autonomy and Longevity, Centre Hospitalier Universitaire d'Angers, 4 rue Larrey, Angers, Pays de la Loire, 49100, France, 33 0241354725; 3 Health Faculty, Université d'Angers, Angers, Pays de la Loire, France; 4 Gérontopôle Autonomie Longévité des Pays de la Loire, Nantes, Pays de la Loire, France; 5 Department of Medical Biophysics, Schulich School of Medicine and Dentistry, Robarts Research Institute, Western University, London, ON, Canada

**Keywords:** spinal puncture, simulation training, patient simulation, augmented reality, user experience, medical education, geriatrics

## Abstract

**Background:**

Lumbar puncture (LP) is a diagnostic procedure in geriatric medicine but may be technically challenging in older adults with spinal deformities, osteoarthritis, frailty, or cognitive impairment. Simulation-based training can provide repeated practice without patient risk. Augmented reality (AR) systems may improve anatomical visualization and sensory feedback, but their added value compared with mannequin-based simulators and whether perceptions differ according to clinical experience remain uncertain.

**Objective:**

This study aimed to compare the user experience (UX) of a traditional mannequin-based LP simulator (M43E; T-S) and an augmented reality simulator with haptic feedback (Sim&Care 2; AR-S) across levels of expertise.

**Methods:**

We conducted a single-center, randomized-order, within-participant comparative UX study at Angers University Hospital, France, in June 2025. A total of 30 participants (n=7, 23.3% sixth-year medical students; n=16, 53.3% postgraduate medical students; and n=7, 23.3% graduated physicians) used both simulators for 15 minutes each, with the exposure order randomized. After each session, participants completed an exploratory 5-point Likert questionnaire assessing face validity (FV), usability, perceived educational value (PEV), and overall satisfaction. After both sessions, they reported simulator preference, reasons for preference, and intention to reuse each simulator; open-ended comments were collected. Dimension-level paired comparisons were performed using the Wilcoxon signed-rank test, expertise-group comparisons used the Kruskal-Wallis test, and categorical outcomes were analyzed using the chi-square test. Item-level analyses were considered exploratory.

**Results:**

The AR-S achieved higher composite scores than the T-S for FV (median 3.8 [IQR 3.6-4.2] vs 3.4 [IQR 3.2-3.8]; *P*=.02) and PEV (median 4 [IQR 3.8-4.6] vs 3.6 [IQR 3.4-4.2]; *P*=.02). No significant differences were observed for usability (median 4.3 [IQR 4-4.95] vs 4.6 [IQR 4.4-4.8]; *P*=.11) or overall satisfaction (median 4 [IQR 4-5] vs 4 [IQR 3-4]; *P*=.16). Exploratory item-level analyses indicated higher ratings for the AR-S for anatomical realism (*P*=.03), tactile sensations (*P*=.003), and perceived acquisition of new knowledge (*P*=.005), whereas the T-S was rated higher for ease of use (*P*=.01), clarity of instructions (*P*=.01), and ease of operation during the procedure (*P*=.003). Overall preference was balanced: 53.3% (16/30) of participants preferred the AR-S, and 46.7% (14/30) of participants preferred the T-S. Preference was not significantly associated with expertise group (*P*=.09), although graduated physicians descriptively favored the AR-S and sixth-year students favored the T-S. In total, 76.7% (23/30) of participants reported willingness to reuse both simulators.

**Conclusions:**

AR and traditional mannequin-based LP simulation appear to offer distinct and complementary educational advantages rather than one modality being uniformly superior. The AR-S was valued for anatomical and sensory realism and PEV, whereas the T-S retained advantages related to procedural familiarity and ease of use with conventional equipment. These findings support tailoring simulator selection to learner expertise and educational objectives. Larger studies incorporating objective procedural performance are needed to determine how the modalities should be integrated into LP curricula.

## Introduction

Lumbar puncture (LP) is a critical procedure in geriatrics, especially for the diagnostic workup of neurodegenerative diseases, including Alzheimer disease and related disorders [1]. However, it is often feared by physicians and medical students due to its invasive nature and its undesirable effects, such as post-LP headaches, bruising, and infections [2]. In older adults, LP may be technically more challenging due to spinal deformities, osteoarthritis, frailty, or behavioral disturbances related to cognitive impairment [3].

Good knowledge of the procedure is the most effective way to avoid adverse effects [4]. Nevertheless, the limited opportunities for medical students to perform LP in clinical settings may contribute to insufficient procedural experience and reduced self-confidence [5]. Notably, research shows that medical students have a higher level of subjective and physiological stress than physicians, affecting their psychomotor capacity and working memory [6], and increasing the risk of post-LP headaches in patients [7].

In France, LP training during medical school remains highly variable depending on the hospital department where students do their internship, the number of students per internship, and the availability of interns and physicians to teach the procedure [8]. The use of simulators, which are becoming more popular, is a promising opportunity to improve traditional LP training. These simulators allow learners to reproduce the main steps of the procedure using the same material and to experience the same sensations, including anatomical landmark palpation, needle insertion, and cerebrospinal fluid collection [8].

Several studies have investigated the benefits of LP simulation training among medical students and residents. They have reported improvements in theoretical knowledge, confidence, and transfer of skills to clinical practice, including higher first-attempt success rates during subsequent LP procedures performed on patients [8-10] and fewer traumatic LPs [11]. However, the benefit of simulation-based training may vary depending on the comparison method and outcome assessed. In a study comparing problem-based learning and simulator-based learning, both approaches improved learner performance, and no significant difference was observed between groups immediately after training [12]. More recently, a randomized controlled trial evaluating virtual reality (VR) training compared with conventional training alone found no significant difference in overall LP performance between groups [13]. In addition, traditional physical simulators have several limitations, including material wear, limited anatomical variability, and restricted representation of tissue biomechanical properties [14]. These limitations may reduce their ability to reproduce the diversity of clinical situations encountered in practice, particularly in older patients, where osteoarthritis and spinal deformities may complicate LP performance [15].

To overcome these limitations, technological advances have led to the development of virtual and augmented reality (AR)–based LP simulators incorporating haptic feedback [15]. While VR-based systems have been more extensively investigated, AR-based LP simulators remain scarce. The Sim&Care Spine simulator (InSimo) is one example of a commercially available AR-based platform designed to provide anatomical visualization and haptic feedback during LP training [16]. This innovative method allows a better visualization of the internal and external anatomy of the patient and has a positive impact on visuospatial capacities, cognitive load, and procedure time [17]. Studies evaluating AR simulators found no significant improvement in LP success rates compared with traditional bedside teaching, but learners reported increased confidence and comfort. Students also reported high educational value, particularly appreciating the haptic feedback and AR visualization [16]. In a study evaluating a mixed reality simulator, participants reported high perceived realism and educational value, particularly regarding anatomical understanding and identification of landmarks. Training with the simulator was also associated with improvements in procedural parameters, including needle positioning, number of attempts, and procedure duration [18].

A comparative study evaluating mixed reality LP training versus traditional high-fidelity mannequin-based simulation showed comparable procedural performance between the 2 approaches. However, mixed reality training was well accepted by learners, with most students considering it to be as effective as traditional simulation and expressing interest in further use of this technology [19]. Although no significant differences were observed in objective outcomes, such as the number of punctures, time to successful puncture, or distance from the target, learners trained with AR reported greater confidence [20]. In addition, VR-based training increased learner engagement, satisfaction, and interest in clinical skills, suggesting that immersive technologies primarily enhance the educational experience rather than procedural performance [13]. AR may enhance student engagement by sustaining learners’ interest and willingness to use this technology beyond the initial training session [21]. Overall, current evidence suggests that AR- and VR-based simulators do not consistently demonstrate superior procedural performance compared with traditional mannequin-based simulation. However, immersive technologies appear to provide specific advantages in terms of anatomical visualization, sensory feedback, learner confidence, engagement, and satisfaction. Although students generally perceive AR simulators as realistic, easy to use, and useful [6,18,22], overall preference remains divided [19]. This suggests that simulator preference may depend on multiple dimensions of user experience (UX), including anatomical and procedural realism, usability, and perceived educational value (PEV). Moreover, these perceptions may vary according to learners’ expertise, as novices and experienced practitioners may value different aspects of simulation, such as procedural familiarity or anatomical understanding.

The objective of this study was therefore to evaluate the UX, including perceived realism, usability, and educational value, of an augmented reality simulator (AR-S; Sim&Care 2) compared with a traditional simulator (T-S; M43E) across different levels of expertise. We hypothesized that participants would prefer the AR simulator for anatomical and sensory realism, while usability and procedural familiarity might favor the traditional model.

## Methods

### Study Design

This study was a single-center, randomized-order, within-participant comparative UX study conducted in the Department of Geriatric Medicine of Angers University Hospital, Angers, France, in June 2025. A single-center design was chosen because the study was conducted within the Angers simulation center, where the T-S was routinely used for training and where the AR simulator was made available for a dedicated evaluation session by the manufacturer. Each participant evaluated both the traditional and AR simulators, with the order of simulator exposure randomized between participants to minimize potential order effects. UX was assessed immediately after each simulator session using a UX questionnaire, followed by a comparative questionnaire after completion of both sessions. This design allowed direct comparison of the 2 simulators while limiting interindividual variability. The study was reported in accordance with the RATE-XR reporting guideline for augmented and VR studies [23].

### Recruitment

Thirty participants were included: 7 (23.3%) graduated physicians, 16 (53.3%) postgraduate medical students, and 7 (23.3%) sixth-year medical students. Participants were recruited through email invitations sent to medical students, residents, and physicians affiliated with Angers University Hospital. Recruitment was voluntary. Inclusion criteria were being a sixth-year medical student, postgraduate medical student, or a graduated physician affiliated with the Department of Geriatric Medicine of Angers University Hospital, involved in medical training or clinical practice, and agreeing to participate in the study. These inclusion criteria were chosen to include participants with different levels of experience with LP. Exclusion criteria included inability to provide informed consent or motor impairment preventing simulator use. The sample size was based on the availability of eligible participants during the study period. No formal sample size calculation was performed because this was an exploratory UX study.

### Ethical Considerations

The study protocol was reviewed and approved by the Ethics Committee of Angers University Hospital (*Comité d’Éthique du Centre Hospitalier Universitaire d’Angers*), which issued a favorable opinion on June 10, 2025 (reference number 2025‐140). The study was conducted in accordance with the principles of the Declaration of Helsinki. Participants received oral information about the study, and their nonobjection to participation was obtained before the beginning of the experiment. The data were pseudonymized using a unique alphanumeric code assigned to each participant to ensure anonymity. No compensation was provided to participants.

### Materials and Protocol

This study was conducted in collaboration with the Angers simulation center (AllSims) and InSimo. The 2 simulators were selected because they represent complementary approaches to LP training: a traditional mannequin-based simulator and an AR-based simulator. The M43E simulator (Kyoto Kagaku) was already available at the Angers simulation center, whereas the Sim&Care 2 simulator (InSimo) was provided by the manufacturer for a dedicated evaluation session.

### Main Characteristics of the Implemented Simulators

The T-S (M43E simulator) is a high-fidelity static LP and epidural procedure simulator designed for procedural training. It consists of an anatomically accurate lumbar region model with a puncture block reproducing relevant structures, including lumbar vertebrae, spinous processes, transverse processes, epidural space, and dura mater. The simulator allows training in landmark identification, needle insertion, and cerebrospinal fluid collection using a real LP needle. It can be positioned in both upright and lateral decubitus configurations, and its translucent puncture block allows visualization of the needle trajectory during the procedure.

The AR-S (Sim&Care 2 simulator) combines a physical interface with virtual anatomical overlay and force-feedback technology delivered via a stylus device and a Microsoft HoloLens 2 headset AR display. The setup consisted of a PC running the simulation software, a Microsoft HoloLens 2 headset for the AR environment, a Touch haptic interface (3D Systems) for force feedback, and a mannequin serving as the physical support for the AR environment. The simulation software provided virtual anatomical visualization and force feedback during needle progression. Sim&Care 2 offers 4 patient cases: standard anatomy, scoliosis, osteoarthritis, and a pediatric case. For each case, users can select between 2 common LP positions: sitting or lateral decubitus. Various customization features enhance the simulation experience, including patient positioning, needle selection, and adjustable difficulty levels. Users can display or hide internal anatomical structures through a transparency mode and activate a visual guidance tool showing the needle trajectory to assist with accurate needle placement within the spinal canal.

### Simulation Protocol

**Figure 1.**
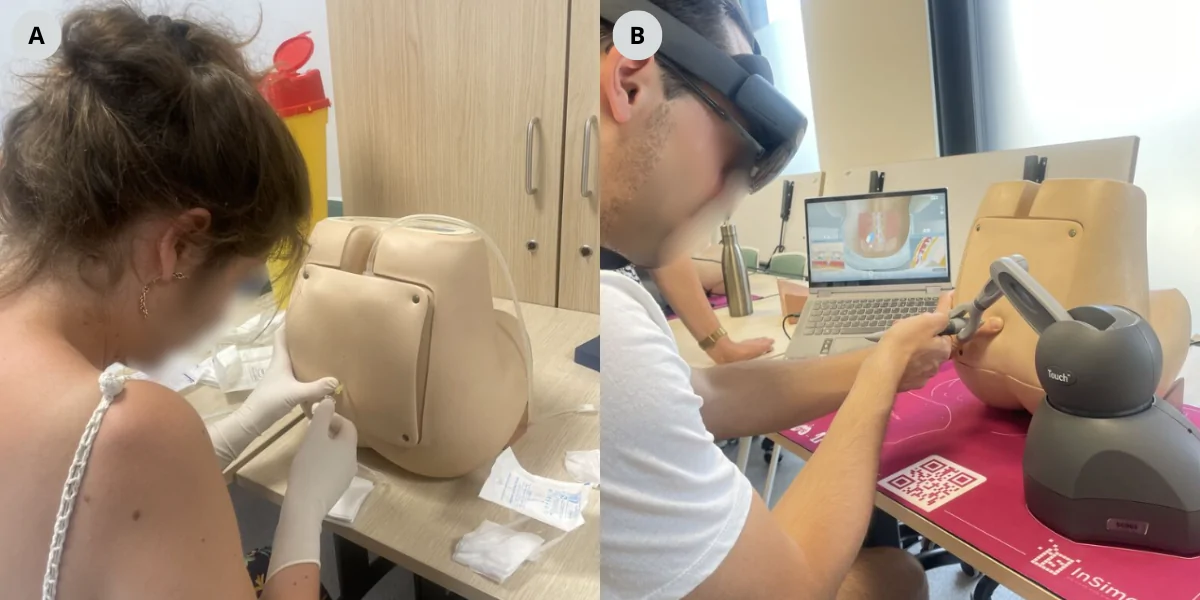
Simulators used during the study sessions. (A) M43E traditional simulator. (B) Sim&Care 2 augmented reality simulator with headset, haptic stylus, and physical interface.

Each participant completed 2 simulation sessions, one with each simulator (Figure 1). Participants were randomized to the simulator exposure order (group A or B). Each session lasted approximately 15 minutes, including a brief familiarization period with the simulator followed by the LP procedure. Participants were allowed to perform several attempts during each session, and the same duration was allocated to both simulators. Only standardized instructions were provided: “Perform a diagnostic lumbar puncture as in clinical practice.” As the objective was to evaluate perceived UX rather than procedural performance, the instructor did not provide corrective feedback or assess technical success during the sessions.

For the AR-S, participants initially performed the procedure using the standard patient configuration. The pediatric patient case was not used in this study. For both simulators, the seated position was used initially during the simulation session, and participants could subsequently practice in the lateral decubitus position if desired.

### Questionnaires

After each simulation session, participants completed a UX questionnaire assessing the simulator they had just used. The questionnaire included 4 dimensions: face validity (FV; 5 items), usability (5 items), PEV (5 items), and overall satisfaction (1 item). Each item was rated on a 5-point Likert scale.

After completing both simulation sessions, participants completed a second comparative questionnaire. This questionnaire assessed overall preference for one of the 2 simulators, the reasons for their preference (realism, ease of use, educational value, sensations, or other), and their intention to reuse each simulator (yes or no). Participants were also invited to provide open-ended comments. Twelve participants provided comments.

### Questionnaire Development

In the absence of a validated LP-specific UX instrument, we developed an exploratory structured questionnaire based on evaluation domains commonly used in simulation-based medical education, including FV [24], usability [25], educational value [26], and satisfaction. No formal validation process or expert assessment was performed before the study due to the exploratory nature of this UX evaluation. Internal consistency was not formally assessed due to sample size limitations. This limitation has been acknowledged in the Discussion section.

FV was assessed using 5 items on a 5-point Likert scale:

- FV1: “The simulator’s anatomy seemed realistic to me.”
- FV2: “The tactile sensations were comparable to a real lumbar puncture.”
- FV3: “Patient positioning was similar to real practice.”
- FV4: “Identification of anatomical landmarks was credible.”
- FV5: “Performing the procedure felt natural.”

Usability was assessed using 5 items on a 5-point Likert scale:

- Usability 1: “The simulator was easy to use.”
- Usability 2: “The instructions for using the simulator were clear.”
- Usability 3: “The simulator was ergonomic.”
- Usability 4: “I easily understood how to operate the simulator during the procedure.”
- Usability 5: “The equipment functioned properly without difficulty.”

PEV was assessed using 5 items on a 5-point Likert scale:

- PEV1: “This simulator is useful for learning lumbar puncture.”
- PEV2: “This simulator could improve my real-life practice.”
- PEV3: “I learned something new with this simulator.”
- PEV4: “The simulator allows progress with training.”
- PEV5: “I would recommend this simulator to other learners.”

Overall satisfaction was assessed using 1 item on a 5-point Likert scale: “I am overall satisfied with this simulator.”

### Data Analysis

Statistical analyses were performed using JASP software. Descriptive analyses were conducted for each questionnaire item, including the median and quartiles. Dimension-level analyses were performed as the primary analysis to align with the study hypothesis. Composite scores were calculated as the mean of the items included in each predefined UX dimension (FV, usability, and PEV). Intraindividual comparisons between T-S and AR-S were performed using the Wilcoxon signed-rank test. Item-level analyses were conducted as exploratory analyses to identify specific differences between the simulators. Given the exploratory nature of these comparisons and the limited sample size, item-level results were interpreted descriptively.

To compare results between the 3 groups based on experience level, the Kruskal-Wallis test was used. Participants’ preferences and their intention to reuse each simulator were analyzed using the Pearson chi-square test. Additionally, a qualitative analysis of the open-ended comments was conducted using a thematic approach.

Two participants submitted incomplete UX questionnaires. Because the missing responses concerned different items, the UX score analyses were based on 29 or 30 participants depending on the item. However, because their comparative questionnaires were fully completed, their responses were included in the analysis of simulator preference and reuse intention.

## Results

### Comparison of UX Dimensions Between Simulators

Results for the UX dimensions are presented in Table 1. Two participants had missing responses for some UX items. Because these missing data concerned different items, the analyses were based on 29 or 30 participants depending on the item. Dimension-level analyses were performed by comparing the composite scores of each predefined UX domain (FV, usability, PEV, and overall satisfaction). The AR-S showed higher scores for FV (*z=*−2.323; *P*=.02) and PEV (*z=*−2.289; *P*=.02) compared with the T-S. No significant differences were observed for the overall usability score (*z=*−1.598; *P*=.11). Finally, for the overall satisfaction item, statistical analysis showed no significant difference in the median overall satisfaction scores between the simulators (*z*=−1.349; *P*=.16).

**Table 1.** Comparison of user experience dimensions between simulators using the Wilcoxon signed-rank test.

Items	M43E, median (IQR)	Sim&Care 2, median (IQR)	*P* value	*r*_pb_ ^a^
Face validity (n=30)	3.4 (3.2-3.8)	3.8 (3.6-4.2)	.02	0.213
Usability (n=29)	4.6 (4.4-4.8)	4.3 (4-4.95)	.11	0.217
Perceived educational value (n=29)	3.6 (3.4-4.2)	4.0 (3.8-4.6)	.02	0.213
Overall satisfaction (n=29)	4.0 (3.0-4.0)	4.0 (4.0-5.0)	.16	0.269

a *r*_pb_: point-biserial correlation coefficient.

### Item-Level Exploratory Analysis

Item-level analyses were conducted as exploratory analyses to identify which specific simulator characteristics contributed to the observed dimension-level differences. These results are presented in Table 2. For the FV items, participants rated the AR-S (Sim&Care 2 simulator) as more realistic on the item “The simulator’s anatomy seemed realistic to me” (FV1), *z*=−2.155; *P*=.03, and on the item “The tactile sensations were comparable to a real lumbar puncture” (FV2), *z*=−2.866; *P*=.003. Concerning the usability items, the T-S (M43E simulator) obtained a significantly higher median on item U1 (“The simulator was easy to use;” *z*=2.456; *P*=.01) and on item U2 (“The instructions for using the simulator were clear;” *z*=2.446; *P*=.01). A highly significant difference was also observed for item U4 (“I easily understood how to operate the simulator during the procedure;” *z*=2.934; *P*=.003), in favor of T-S. For the PEV items, statistical analysis showed a significant difference for item PEV3 (“I learned something new with this simulator”), with a higher median for the AR-S (*z*=−2.808; *P*=.005).

**Table 2.** Comparison of user experience items between simulators using the Wilcoxon signed-rank test.

Items	M43E, median (IQR)	Sim&Care 2, median (IQR)	*P* value	*r*_pb_ ^a^
FV^b^1 (n=30)	3 (3-4)	4 (3.25-5)	.03	0.244
FV2 (n=30)	3 (2-4)	4 (3.25-4)	.003	0.225
FV3 (n=30)	4 (3-4)	4 (3-4)	.10	0.328
FV4 (n=30)	4 (3-4)	4 (3-4)	.42	0.269
FV5 (n=30)	4 (3-4.75)	4 (3-4)	.65	0.244
Usability 1 (n=30)	5 (4-5)	4 (3.25-5)	.01	0.277
Usability 2 (n=30)	5 (5-5)	5 (4-5)	.01	0.342
Usability 3 (n=30)	4 (4-5)	4 (4-5)	.83	0.25
Usability 4 (n=29)	5 (5-5)	5 (4-5)	.003	0.328
Usability 5 (n=29)	5 (3-5)	5 (4-5)	.15	0.277
PEV^c^1 (n=29)	4 (4-5)	4 (4-5)	.92	0.269
PEV2 (n=29)	3.1 (3-5)	4 (4-5)	.47	0.256
PEV3 (n=29)	2.5 (2-3)	4 (3-4)	.005	0.239
PEV4 (n=29)	4 (3-5)	4 (4-5)	.45	0.256
PEV5 (n=29)	4 (4-5)	4 (4-5)	.45	0.269

a *r*_pb_: point-biserial correlation coefficient.

b FV: face validity.

c PEV: perceived educational value.

### Group Effect

Results for group differences are presented in Table 3. The “group” factor showed a significant effect on one realism item for the AR-S, FV4 (“Identification of anatomical landmarks was credible;” Kruskal-Wallis test, *P*=.04), with a lower median among graduated physicians. For the T-S, the “group” factor showed a significant effect on the realism item FV3 (“Patient positioning was similar to real practice;” Kruskal-Wallis test, *P*=.048), with a higher median among medical students.

**Table 3.** Effect of participant group on user experience questionnaire items for the M43E and Sim&Care 2 simulators using Kruskal-Wallis test.

Items	M43E, *P* value	Sim&Care 2, *P* value
FV^a^1	.12	.95
FV2	.25	.43
FV3	.048	.06
FV4	.20	.04
FV5	.65	.52
Usability 1	.92	.72
Usability 2	.14	.83
Usability 3	.05	.25
Usability 4	.19	.81
Usability 5	.89	.22
PEV^b^1	.99	.42
PEV2	.11	.10
PEV3	.93	.68
PEV4	.37	.15
PEV5	.29	.38
Overall satisfaction	.22	.37

a FV: face validity.

b PEV: perceived educational value.

### Participants’ Preference and Intention to Reuse

Among the 30 participants, 16 (53.3%) reported a preference for the AR-S, while 14 (46.7%) preferred the T-S. Among graduated physicians, 85.7% (6/7) preferred the AR-S. Among sixth-year medical students, 71.4% (5/7) preferred the T-S. For postgraduate medical students, the 2 simulators were equally preferred (8/16, 50% each). However, simulator preference was not significantly associated with participant group (*χ*^2^_2_=4.8; *P*=.09).

The reasons given in favor of the AR-S mainly concerned “better sensations” (11/30, 36.7%) and greater educational value (10/30, 33.3%). For the T-S, the most frequently cited arguments were greater realism (6/19, 31.6%) and greater ease of use (5/19, 26.3%).

Regarding the intention to reuse, 76.7% (n=23) of participants were willing to use both simulators again. A total of 10% (n=3) were willing to reuse only the AR-S, and the same proportion were willing to reuse only the T-S. Finally, 3.3% (n=1) did not intend to use an LP simulator again. There was no association between the intention to reuse the simulators and participant group (*χ*^2^_6_=8.0; *P*=.24).

## Discussion

### Principal Findings

The aim of this study was to compare the UX of sixth-year second cycle medical students, postgraduate medical students, and graduated physicians using 2 LP simulators, a traditional one (M43E simulator) and an AR one (Sim&Care 2 simulator). Our hypothesis was that participants would prefer the AR model due to its greater realism and perceived pedagogical value. However, our hypothesis was also that these results could be influenced by the ease of use of the traditional model and the realism of the procedure, particularly due to the use of an actual LP needle. Overall, our findings partially confirmed this hypothesis. Dimension-level analyses showed that the AR simulator was associated with higher perceived FV and PEV compared with the T-S. However, no significant differences were observed for usability or overall satisfaction. To further understand the differences observed between simulators, complementary item-level analyses and qualitative data were examined to identify specific characteristics that may explain participants’ perceptions.

Regarding anatomical and technical realism, participants in the study reported higher scores for the AR-S. It was perceived as having greater anatomical realism and providing sensations comparable to those experienced in real life. In the open-ended comments, 2 participants noted that the AR-S offered a more pleasant and realistic sensation during ligament passage and needle insertion. However, statistical analyses did not show a significant difference between the simulators for item FV5 (“Performing the procedure felt natural”). To explain this, participants reported that the stylus of the AR-S did not provide the same handling sensation as a real LP needle, mainly because of its larger diameter than that of the needle used with the T-S. Among groups, graduated physicians gave lower scores to the AR-S on the item “Identification of anatomical landmarks was credible,” which may be explained by their greater expertise. Conversely, sixth-year second cycle medical students gave higher scores for both simulators on FV3 (“Patient positioning was similar to real practice”), which may be due to their limited clinical experience.

Regarding usability, no significant difference was observed between the 2 simulators at the overall dimension level. However, item-level analyses and participants’ comments suggested some practical advantages for the T-S. This may be partly explained by participants’ prior exposure to the T-S during their medical training, which may have made them more familiar with its use. Participants who preferred the T-S cited ease of use and realism as reasons, as it is simpler to set up and the procedure is closer to real-life practice. However, 2 residents noted difficulties in locating anatomical landmarks (especially for osteoarthritis simulation) and noted mannequin wear at landmark areas—issues inherent to this type of simulator compared with AR-based simulators.

Regarding PEV, participants in the study reported higher scores for the AR-S. However, item-level analysis showed a higher score for the AR-S on PEV3 (“I learned something new with this simulator”). In the open-ended comments, 1 resident described the AR-S as an “excellent learning tool,” that provided a better 3D representation of anatomy than the T-S.

However, preferences were split (16/30, 53.3% preferred the AR-S and n=14, 46.7% preferred the T-S). Descriptively, preferences differed across participant groups: graduated physicians mostly preferred the AR model, and sixth-year second cycle medical students preferred the T-S. These results may be explained by experience: medical students, having rarely or never performed an LP, may value a simulator that is easy to use and provides realistic procedural execution (including needle handling) during their initial LP training. Graduated physicians, with expertise in the procedure, preferred the AR-S, which may have allowed them to better appreciate its sensations and anatomical realism. Regarding the intention to reuse the simulators, results were similar for both, showing no marked preference.

These results are consistent with and complementary to the existing literature comparing traditional and immersive simulation for LP training. While several studies have shown that AR, mixed reality, or VR simulators improve learner confidence, engagement, satisfaction, and anatomical understanding, they generally report procedural performance comparable to that obtained with conventional mannequin-based simulation rather than clear superiority of immersive technologies [13,16,18,19]. Our results complement these findings by showing that these technologies are not necessarily perceived as superior across all dimensions of the user experience. Instead, participants appeared to distinguish between different aspects of realism and educational value. The AR-S was particularly valued for anatomical visualization, sensory realism, and understanding of anatomical landmarks, whereas the T-S remained preferred for procedural realism and ease of use. These findings may explain why previous studies reported high satisfaction with immersive simulators despite the absence of consistent improvements in procedural performance.

Similar findings have been reported in other fields of medical simulation, where students preferred the T-S for learning clinical procedures, despite considering virtual simulators more enjoyable and immersive [27].

These observations suggest that AR-S and T-S should be considered complementary rather than competing educational tools. Traditional mannequin-based simulation may remain particularly appropriate for novice learners, who need to become familiar with real equipment and procedural workflow. In contrast, AR simulation may provide additional value for more advanced learners by enhancing anatomical understanding, spatial orientation, and sensory realism, especially in anatomically complex situations such as those frequently encountered in older patients. In this way, complementary use of both approaches may be considered to combine traditional mannequin simulations, which allow the use of real medical equipment particularly for novice medical students, with AR simulations, which offer dynamic scenarios and more realistic sensations for postgraduate medical students or graduated physicians who already have substantial experience with LPs.

This study has some limitations. First, the sample size limited the statistical power of subgroup analyses and the generalizability of the findings across different levels of expertise [28]. Second, some participants, particularly residents and graduated physicians, had previous experience with the T-S, which may have influenced their perception of usability and realism in favor of the T-S. Similarly, previous LP experience was not systematically collected. This may have affected participants’ perceptions of realism and educational value, particularly among medical students with limited exposure to the procedure. Third, some dimensions of realism were difficult to capture using Likert-scale evaluations. While participants perceived the AR-S as more realistic in terms of anatomical visualization and sensory feedback, some considered the procedural performance less realistic due to the use of a stylus rather than conventional LP equipment. This highlights the multidimensional nature of realism in simulation-based education. In addition, PEV may have varied according to participants’ level of expertise. Novice learners may value simulators for acquiring basic procedural knowledge, whereas more experienced participants may assess educational value according to more advanced objectives, such as refining procedural skills or managing complex clinical situations. Furthermore, some items may overlap between UX dimensions, reflecting the multidimensional nature of user experience assessment. Finally, multiple item-level comparisons increase the risk of type I error, and these results should therefore be interpreted as exploratory.

Despite these limitations, this study provides insight into how different levels of expertise may influence simulator perception and highlights the importance of considering specific educational objectives when selecting simulation modalities. Beyond the technology itself, the educational value of immersive simulation also depends on its integration into the curriculum, with learning activities adapted to learners’ levels of expertise and educational objectives [29]. Future studies with larger cohorts and objective performance assessments are needed to determine how these complementary simulation modalities can be integrated into LP training pathways.

### Conclusions

Our hypothesis was partially confirmed. The AR-S was perceived as providing greater anatomical and sensory realism, while the T-S remained valuable for procedural familiarization using real equipment. However, these differences did not translate into an overall preference for the AR-S.

These findings suggest that LP simulation should not rely on a single modality but rather on selecting the appropriate simulator according to learners’ levels of expertise and educational objectives. Traditional mannequin-based simulation remains relevant for novice learners who need to develop procedural skills and familiarity with real equipment, whereas AR simulation may provide additional value for more advanced learners by enhancing anatomical understanding and sensory feedback.

Future research should evaluate how these complementary simulation modalities can be optimally integrated into medical curricula and assess their impact on procedural performance, learner confidence, and ultimately patient care.

## Abbreviations

AR: augmented reality
AR-S: augmented reality simulator
FV: face validity
LP: lumbar puncture
PEV: perceived educational value
T-S: traditional simulator
UX: user experience
VR: virtual reality
